# Attitudes towards people with mental disorders: results of a psychometric evaluation and confirmatory factor analysis of the stigma towards people with mental disorders (SToP-MD) scale

**DOI:** 10.1186/s40359-026-04627-x

**Published:** 2026-05-01

**Authors:** Jan Christopher Cwik, Marcella L. Woud, Simon E. Blackwell, Tobias Teismann, Ines Keita, Jürgen Margraf

**Affiliations:** 1 Professor of Applied Psychology, Faculty of Health Care Research, Hochschule Niederrhein, Reinarzstraße 49, Krefeld, 47805 Germany; 2 Department of Clinical Psychology and Experimental Psychopathology, Institute of Psychology, University of Göttingen, Göttingen, Germany; 3 Mental Health Research and Treatment Center, Faculty of Psychology, Ruhr- Universität Bochum, Bochum, Germany; 4 German Alliance against Depression, Leipzig, Germany

**Keywords:** Stigma, Attitudes, Mental disorders, Scale, Assessment

## Abstract

Stigmatizing attitudes toward individuals with mental disorders represent a major barrier to treatment, recovery, and social inclusion. The present research introduces and psychometrically evaluates the German-language Stigma Toward People with Mental Disorders scale (SToP-MD) across three independent studies with distinct samples.

In study 1 (*N* = 266), an initial item pool was developed and refined based on theoretical frameworks and exploratory factor analysis. In study 2 (*N* = 448), confirmatory factor analysis supported a two-factor structure comprising prejudiced stigmatization (SToP-MD-PS) and assumption of problems (SToP-MD-AP). The model demonstrated adequate fit according to conventional indices (CFI = 0.97, TLI = 0.96, SRMR = 0.07), although robust indices indicated only moderate fit (robust CFI = 0.91, robust RMSEA = 0.13). Internal consistency was good for the PS subscale (ω = 0.83) but limited for the AP subscale (ω = 0.51). In study 3 (*N* = 266), the scale’s sensitivity to short-term change was examined following exposure to differently framed media content.

As hypothesized, the SToP-MD subscales were positively associated with depression stigma (DSS) and social distance (SDI), and negatively correlated with openness and agreeableness (NEO-FFI), supporting convergent validity. Discriminant validity was partially confirmed by low or non-significant correlations with attitudes toward physically disabled individuals (ATDP), suicide-related cognitions (CCSS), and socially desirable responding (BIDR).

Across all three studies, the SToP-MD demonstrated preliminary yet consistent evidence of structural and construct validity, as well as change sensitivity. It captures both overt prejudices and implicit burden assumptions, offering a nuanced assessment of public stigma toward mental disorders. The scale can serve as a valuable tool in stigma research, public health monitoring, and evaluation of interventions. Future research should extend validation to more diverse samples and test predictive and longitudinal utility.

**Supplementary Information:**

The online version contains supplementary material available at 10.1186/s40359-026-04627-x.

## Introduction

The stigmatization of people with mental disorders represents a major public health concern, as it negatively affects help-seeking, treatment adherence, recovery, and social inclusion [1–4]. Stigma is commonly conceptualized as a social process involving labeling, stereotyping, separation, status loss, and discrimination within contexts of unequal power [5–7]. In this sense, public stigma reflects negative attitudes and beliefs held by the general population toward individuals with mental disorders, often emphasizing dangerousness, unpredictability, or personal responsibility.

A substantial body of research has investigated stigma and its consequences, as well as sociodemographic and psychological correlates [2–4, 8–10]. However, despite the growing number of studies, the assessment of stigma remains methodologically challenging. Existing instruments differ considerably in their conceptual focus and often assess either self-stigma, perceived public stigma, or disorder-specific attitudes (e.g., toward depression or schizophrenia) [11–14]. Moreover, several widely used scales are lengthy, conceptually heterogeneous, or rely on comparisons with “average” individuals, which may introduce ambiguity and susceptibility to social desirability biases [15].

Although more recent instruments have improved the assessment of stigma (e.g., the Opening Minds Scale for Health Care Providers [OMS-HC; [16]], the Opening Minds Workplace Attitudes Scale [OMS-WA; [17]], the Attribution Questionnaire [AQ-27; [18]], or the Prejudice towards People with Mental Illness scale [PPMI; [19]]), important limitations remain. Many measures focus on specific populations (e.g., health care providers or workplace settings), target specific disorders, or assess only selected components of stigma, such as social distance or attributional beliefs. Consequently, brief and generalizable instruments that capture public stigma toward mental disorders as a broad category are still limited.

At the same time, research has demonstrated that public stigma varies across mental disorders, with conditions such as schizophrenia often being associated with higher levels of perceived dangerousness than depression [20–22]. While disorder-specific measures are therefore valuable, a generalizable approach also has merit. Assessing stigma toward “mental disorders” as a broader category allows for the investigation of overarching attitudes that may shape public discourse, policy preferences, and responses to mental health-related events. However, such an approach may also obscure disorder-specific differences, representing a potential limitation that should be considered when interpreting results.

Building on these considerations, the present research aimed to develop a brief, theory-informed instrument for assessing public stigma toward people with mental disorders in general. The Stigma Towards People with Mental Disorders (SToP-MD) scale was designed to capture two central dimensions of stigma: (1) prejudiced evaluations reflecting negative stereotypes (e.g., dangerousness, incompetence), and (2) assumptions about persistent personal and societal burden associated with mental disorders. In contrast to many existing scales, the SToP-MD focuses on public stigma-related attitudes within the general population and is intended to provide a concise and broadly applicable measure suitable for research and intervention contexts.

The present research comprises three studies. Study 1 examined the factorial structure, reliability, and construct validity of the SToP-MD using exploratory factor analysis. Study 2 tested the proposed factor structure in an independent sample using confirmatory factor analysis. Study 3 investigated the scale’s sensitivity to short-term changes in stigmatizing attitudes following exposure to different types of media content. Together, these studies provide an initial evaluation of the psychometric properties and potential applications of the SToP-MD scale.

## Study 1: exploratory factor analysis and psychometric properties of the SToP-MD scale

### Methods

#### Participants and procedure

The study was conducted using an online questionnaire developed using SoSci Survey [23] and made available to participants on www.soscisurvey.com. Participants were recruited online, completed the survey anonymously, and provided informed consent prior to participation.

The complete sample consisted of *N* = 335 participants. Because of incomplete questionnaires, 69 participants were excluded, so the final sample consisted of *n* = 266 (74.9%) participants, of whom 69.9% (*n* = 186) were female, and 30.1% (*n* = 80) were male. Age ranged from 18 to 88 years, with a mean of 28.77 years (*SD* = 13.69). The employment status of the participants was as follows: 152 (57.1%) students, 55 (20.7%) workers/employees, 19 (7.1%) clerks, 11 (4.1%) trainees, 10 (3.8%) pupils, 8 (3.0%) freelancers, 6 (2.3%) retired participants, and 5 (1.9%) job-seeking participants. In terms of nationality, 262 (98.5%) were German, 1 (0.4%) was Austrian, 1 (0.4%) was Spanish, 1 (0.4%) was Brazilian, and 1 (0.4%) was a US-American citizen. The educational background of participants was as follows: 8 (3.0%) pupil, 1 (0.4%) certificate of secondary education, 5 (1.9%) secondary school level I certificate, 21 (7.9%) apprenticeship, 16 (6.0%) vocational diploma, 154 (57.9%) diploma from German secondary school qualifying for university admission or matriculation, 57 (21.4%) polytechnic degree or university degree, and 4 (1.5%) participants had another educational background. Of all participants, 48 (18.0%) had no own income, 133 (50.0%) had a low income (< 1000 €), 62 (23.3%) participants had a middle income (1000 € − 3000 €), 18 (6.8) participants had a high income (< 3000 €), and 5 (1.9%) participants did not want to give any information about their income.

Overall, 56 (21.1%) participants stated that they have or have had contact with a mental health professional, 19 (7.1%) reported suffering from a mental disorder at the time of their participation in the study, 42 (15.8%) reported having suffered from a mental disorder in the past, 73 (27.4%) participants stated that a loved one was suffering from a mental disorder at the time of their participation, and 74 (27.4%) stated that a loved one had suffered from a mental disorder in the past.

#### Construction of the stigma towards people with mental disorders scale

The construction of the SToP-MD scale comprised five steps. In the first step, we screened the stigma scales listed in the reviews by Sibley and Duckitt [24] and by Brohan et al. [1] to identify relevant aspects of stigmatization. In the second step, the psychological literature on stigmatization, prejudice, stereotypes, and attitudes was reviewed, with particular attention to the stereotype dimensions identified by Hayward and Bright [25].

In the third step, items were generated based on this information, reports from users of mental health services (*N* = 20), clinical experience, and public discourse surrounding the Germanwings disaster [26–28]. Item development followed an iterative, expert-informed process. Initial item drafts were discussed with licensed psychotherapists who were either employed as research staff at a university department of clinical psychology and psychotherapy or involved in providing outpatient psychotherapeutic care within a university training clinic. In addition, master’s students in clinical psychology and discussion groups with patients with lived experience of mental disorders contributed to the refinement of the item pool. Patient discussions were used to ensure that the selected content reflected experiences and concerns commonly encountered in everyday social contexts.

Across these stages, feedback primarily focused on the conceptual content the items should capture, including which aspects of stigmatizing attitudes toward people with mental disorders were most relevant to represent. The process emphasized content coverage rather than formal evaluations of item clarity or redundancy, and no formal cognitive pretesting was conducted. In the final steps, items were reviewed for redundancy and reformulated to yield sufficient variance in participants’ responses.

The first draft of the SToP-MD scale consisted of 22 items measuring stigmatization towards people with mental disorders in general (see Table 1), on a six-point Likert scale ranging from 1 = “completely disagree” to 6 = “completely agree”. The scale was constructed to measure stigmatization on several aspects of everyday life, for instance familial aspects (e.g., “A family member with a mental disorder often entails many burdens for the family.”), occupational aspects (e.g., “I would have no hesitation to work with a person with a mental disorder.”) or aspects concerning society (e.g., “People with mental disorders are burdensome for society.”). Additionally, prejudices against people with mental disorders (e.g., “Many people with mental disorders are less intelligent.”) or against mental disorders by their nature (e.g., “Mental disorders are often a sign of a weakness of character.”) were considered. The polarity of four items (2, 7, 17, 20) must be reversed so that a higher sum score indicates more negative attitudes towards people with mental disorders.

**Table 1:** Original 22 items of the Stigma Towards People with Mental Disorders (SToP-MD) scale in German and the English translation of these items

Item #	Item (German original version)	Item (English translation)
1	Meiner Meinung nach wäre es besser, wenn Personen mit psychischen Störungen nur in Jobs mit geringer Verantwortung arbeiten dürften.	In my opinion it would be better if people with mental disorders could only work in jobs with less responsibility.
2	Personen mit psychischen Störungen sind beruflich genauso leistungsfähig wie Personen ohne psychische Störungen.	People with mental disorders are as efficient professionally as people without mental disorders.
3	Ein Familienmitglied mit einer psychischen Störung bringt oftmals viele Belastungen für die Familie mit sich.	A family member with a mental disorder often poses a great burden to the family.
4	Personen mit psychischen Störungen belasten die Gesellschaft.	People with mental disorders are burdensome for society.
5	Wenn ein Mitglied meiner Familie an einer psychischen Störung leiden würde, wäre mir das unangenehm.	If a member of my family were to suffer from a mental disorder, this would be unpleasant for me.
6	Personen mit psychischen Störungen werden häufiger straffällig.	People with mental disorders are more delinquent.
7	Ich hätte keine Bedenken, mit einer Person mit einer psychischen Störung zusammenzuarbeiten.	I would have no hesitation to work with a person with a mental disorder.
8	Man sollte zum Schutz anderer die Schweigepflicht von PsychotherapeutInnen und PsychiaterInnen überdenken.	One should protect others by reconsidering the non-disclosure obligation of psychotherapists and psychiatrists.
9	Personen mit psychischen Störungen verursachen häufig unverhältnismäßig hohe Kosten für das Gesundheitssystem.	People with mental disorders often cause disproportionately high costs for the health system.
10	Manchmal denke ich, dass eine psychische Störung nur eine vorgeschobene Entschuldigung ist.	Sometimes I think that a mental disorder is only an excuse for something else.
11	Es ist am besten, Personen mit psychischen Störungen einfach aus dem Weg zu gehen.	It is best just to get out of the way of people with mental disorders.
12	Personen mit psychischen Störungen sind oft unberechenbar.	People with mental disorders are often unpredictable.
13	Psychische Störungen sind häufig auch ein Zeichen von Charakterschwäche.	Mental disorders are often a sign of a weakness of character.
14	Personen mit psychischen Störungen haben eher Probleme, soziale Regeln zu befolgen.	People with mental disorders have more problems following social rules.
15	Viele Personen mit psychischen Störungen sind weniger intelligent.	Many people with mental disorders are less intelligent.
16	Oftmals sind Personen mit psychischen Störungen selbst schuld an ihren Problemen.	People with mental disorders are often to blame for their problems.
17	Ich denke nicht, dass Personen mit psychischen Störungen ihre Arbeitskollegen belasten.	I do not think that people with mental disorders burden their work colleagues.
18	Wer einmal unter einer psychischen Störung leidet, wird wahrscheinlich immer Probleme damit haben.	Someone who has once suffered from a mental disorder, is likely to always have problems with it.
19	Aufgrund der häufigeren Krankheitstage sollte in Betracht gezogen werden, dass Personen mit psychischen Störungen höhere Krankenkassenbeiträge zahlen.	Due to the frequent sick days, the possibility that people with mental disorders pay higher health insurance contributions should be considered.
20	Personen mit psychischen Störungen sind eher keine Gefahr für andere.	People with mental disorders are most likely no threat to others.
21	Würde ich selber eine psychische Störung entwickeln, würde ich das vermutlich vor anderen verbergen.	If I were to develop a mental disorder, I would probably conceal it from others.
22	Personen mit psychischen Störungen können einem Angst machen.	People with mental disorders can cause fear in others.

Item development was guided by the stigma framework proposed by Link and Phelan [6]. Items were constructed to capture two theoretically relevant components: prejudiced evaluations emphasizing dangerousness and incompetence, and assumptions about persistent personal and societal burden associated with mental disorders.

#### Measures

All scales used in this study are established and previously published. The sources for each scale are listed in the following section, along with the description of each scale.

#### Depression Stigma Scale (DSS)

The DSS [29] assesses stigmatizing attitudes towards people with depression and consists of 18 items that are answered on a five-point Likert scale, ranging from 1 = “strongly disagree” to 5 = “strongly agree” [14]. The DSS comprises two subscales measuring personal stigma (DSS-PS) and perceived stigma (DSS-PC), each with 9 items. In this context, “personal stigma” refers to respondents’ own attitudes toward people with depression. The stigma score is calculated by summing up all item scores, with a potential maximum score of 90. Higher scale scores indicate a higher manifestation of stigmatization towards persons with depression. The internal consistency of the total scale is α = 0.78, whereas the DSS-PS subscale has an internal consistency of α = 0.76–0.77, and the DSS-PC subscale of α = 0.82 [14, 30]. We expected significantly positive associations between DSS and SToP-MD, and thus this questionnaire was used for establishing convergent validity of the SToP-MD scale.

#### Social Distance Items (SDI)

The SDI was used by Link et al. [31] to measure social distance after reading case vignettes describing a person with a mental disorder or physical problems and divergent levels of behavior. The SDI has excellent internal consistency α = 0.92. Compared to the original version of the scale that uses a four-point Likert scale format, we used a five-point Likert scale from 1 = “definitely willing” to 5 = “definitely unwilling” referring to [32]. By summing scores up, one receives a score for social distance whereby higher scores represent higher social distance from people with mental disorder. The SDI was also used for establishing convergent validity of the SToP-MD scale. Thus, we expected significantly positive associations between both scales.

#### NEO Five Factor Inventory (NEO-FFI)

The NEO-FFI [33] is a self-rating scale, which assesses the factors neuroticism, extraversion, openness to experience, agreeableness, and conscientiousness with each 12 items on a five-point Likert scale from “strongly disagree” to “strongly agree”. In the present study only the three subscales for neuroticism (NEO-FFI-N), openness to experience (NEO-FFI-O), and agreeableness (NEO-FFI-A) of the German version [34] were used. Lower scores in NEO-FFI-N are associated with emotional stability, whereas higher scores are associated with emotional instability. Lower scores in NEO-FFI-O are associated with being more conservative and careful, whereas higher scores are associated with curiosity. Finally, lower scores in NEO-FFI-A are associated with competition orientation and antagonism, whereas higher scores are associated with being more altruistic. The internal constancy of this German three scales lies between α = 0.63 (openness to experience) and α = 0.82 (neuroticism), with a test-retest-reliability between r_tt_ = 0.67 (openness to experience) and α = 0.82 (neuroticism) [35, 36]. Additionally, factor analyses and correlation analyses with scales measuring similar and different constructs confirm the validity of the questionnaire [34]. We expected that openness to experience and agreeableness would reveal significantly positive associations with the SToP-MD scale, whereas we hypnotized a significantly negative association between SToP-MD and neuroticism.

#### Attitudes Toward Disabled Persons scale (ATDP)

The ATDP scale [37] measures negative attitudes towards people who are physically disabled. In our study we used the German version of the scale (EKB; Einstellung gegenüber Körperbehinderten), which is similar to the [38]. ATDP measures attitudes towards people who are physically disabled on four subscales, with an internal consistency (Cronbach’s α) between α = 0.88 and α = 0.93 as well as adequate factorial structure and validity [38, 39]. For the present study subscales for “discomfort and insecurity in the presence of physically disabled persons” (ATDP -D; 15 items) and “rejection of social integration” (ATDP -R; 6 items) were used. Whereas Seifert and Bergmann [38] report good internal consistency for the first subscale (α = 0.81–0.90), the internal consistency for the latter is questionable (α = 0.56–0.77). Items of the ATDP are rated on a six-point Likert scale from 1 = “completely disagree” to 6 = “completely agree”. Most of the items became reoriented (items: 1, 2, 3, 5, 6, 7, 8, 10, 12, 13, 14, 16, 18, 19), thus higher sum scores indicate higher adverse attitudes towards people physically disabled persons. We expected no significant associations between the ATDP and the SToP-MD scale, on the basis that attitudes toward physically disabled people may be based on a different psychological construct to attitudes towards people with mental disorders. Thus, this questionnaire was used to investigate divergent validity.

#### Cognitions Concerning Suicide Scale (CCSS)

The German version of the CCSS [40] is a 17-item self-report measure to assess attitudes towards suicide [41]. All items are answered on a six-point Likert scale ranging from “0 = I disagree” to “5 = I agree”. To receive a consistent scoring of the questionnaire, scores of 8 items are reversed (items: 4, 5, 9, 10, 11, 12, 14, 17), such that higher scores indicate a more positive view of suicide. The CCSS consists of three subscales measuring the “right to commit suicide” (CCSS-S: 8 items; e.g., “Everyone has the right to commit suicide”, “When life consists of intolerable pain, suicide is an acceptable alternative”), suicide as an “interpersonal gesture” (CCSS-I: 5 items, e.g., “I sometimes think suicide would be a good way to pay back people who have hurt me deeply”; “Taking my own life would be a good way to make sure I would always be remembered”), and a third factor measuring “resiliency” (CCSS-R: 4 items; “Even if I got tired of living, I would not seriously consider suicide as a way out”; “Even if I could not be with the person I love, I would not consider suicide”). The German version of the CCSS has a sufficient internal consistency for the overall CCSS-scale (α = 0.83) and all three subscales (CCSS-S: α = 0.83; CCSS-I: α = 0.70; CCSS-R: α = 0.67) as well as an acceptable construct and discriminant validity [40]. The CCSS was also used to investigate divergent validity. We did not expect any significant association between the CCSS and the SToP-MD scale.

#### Balanced Inventory of Desirable Responding (BIDR)

The BIDR [42] assesses self- and other-deception on two scales by each 20 seven-point Likert scaled items. Both scales are balanced between negatively and positively oriented items. For the present study, we used the German 20-items version of the BIDR [43]. All items are anchored from 1 = “completely disagree” to 7 = “completely agree”. The German version contains 13 negatively oriented items. Consequently, thirteen items are reverse-scored (items: 2, 4, 5, 7, 9, 10, 11, 12, 14, 15, 17, 18, 20). Summing all items provides a score for social desirability. Both scales of the German version showed acceptable internal consistencies (self-deception: α = 0.64; other-deception: α = 0.66), clear factor structure, and good construct validity [43]. This questionnaire was used to control for the effects of social desirability on participants’ answers. As social desirability represents a general response tendency rather than a specific attitudinal domain, low or non-significant associations with the SToP-MD scale were anticipated, which would support the scale’s divergent validity. 

#### Statistical analyses

In a first step, we conducted an item analysis to investigate which items of the initial version of the SToP-MD scale should be retained. We excluded items with an item difficulty < 30.0%.

The factorial structure of the SToP-MD scale was examined using exploratory factor analysis (EFA) with principal axis factoring based on a polychoric correlation matrix and oblimin rotation. Sampling adequacy was assessed using the Kaiser–Meyer–Olkin measure (KMO) [44] and Bartlett’s test of sphericity [45]. Factor retention was evaluated using parallel analysis based on polychoric correlations.The correlation between the SToP-MD scales was analyzed using the Spearman correlation coefficient (*r*_*S*_).

Calculating McDonald’s ω [46–48] determined the internal consistency of the derived scales. Kurtosis, skewness, and means were calculated, and the normal distribution of the sum scores of all scales used in this study was tested using the Kolmogorov-Smirnov test. Finally, construct validity was investigated using *r*_*S*_ between the SToP-MD subscales, demographic variables, and criterion measures. For the association between the SToP-MD subscales and nominal demographic variables, eta was calculated.

Interpretation of the effect sizes was based on Cohen [49], where *r*_*s*_ = 0.1–0.29 represents a small association, *r*_*s*_ = 0.3–0.49 represents a medium association, and *r*_*s*_ ≥ 0.5 represents a large association. Associations between SToP-MD scales and nominal demographic variables were analyzed by using cross-tabulations and Χ^2^-tests. Data analysis was conducted using R [version 4.5.2; [50]].

### Results

#### Item Selection

In a first step, an item analysis was conducted to investigate the adequacy of all 22 items included in the questionnaire. Therefore, item difficulty was calculated.

Subsequently, nine items with inadequate item difficulty (< 30.0%) were excluded. Furthermore, three items with negative polarity were also excluded because of marginal benefits but considerable disadvantages for the psychometric properties and dimensionality of the scale [51]. Thus, the final version of the SToP-MD scale comprises 10 items. The item difficulty of remaining items ranged from 30.0% to 72.0% (see Table 2).

**Table 2:** Results of reliability and item analysis of the initial items of the Stigma Towards People with Mental Disorders Scale (SToP-MD) (Study 1; *N* = 266)

Item #	M (SD)	range (min – max)	item difficulty (%)	action
SToP-MD 1	2.94 (1.55)	1–6	38.8	
SToP-MD 2	3.50 (1.39)	1–6	50.0	item deleted ^b^
SToP-MD 3	4.60 (1.9)	1–6	72.0	
SToP-MD 4	2.66 (1.38)	1–6	33.2	
SToP-MD 5	2.45 (1.60)	1–6	29.0	item deleted ^a^
SToP-MD 6	2.24 (1.35)	1–6	24.8	item deleted ^a^
SToP-MD 7	2.36 (1.40)	1–6	27.2	item deleted ^a^
SToP-MD 8	2.84 (1.71)	1–6	36.8	
SToP-MD 9	2.50 (1.43)	1–6	30.0	
SToP-MD 10	2.16 (1.51)	1–6	23.2	item deleted ^a^
SToP-MD 11	1.61 (1.23)	1–6	12.2	item deleted ^a^
SToP-MD 12	3.23 (1.35)	1–6	44.6	
SToP-MD 13	1.77 (1.30)	1–6	15.4	item deleted ^a^
SToP-MD 14	2.76 (1.39)	1–6	35.2	
SToP-MD 15	1.47 (1.04)	1–6	9.4	item deleted ^a^
SToP-MD 16	1.77 (1.21)	1–6	15.4	item deleted ^a^
SToP-MD 17	3.25 (1.32)	1–6	45.0	item deleted ^b^
SToP-MD 18	3.17 (1.44)	1–6	43.4	
SToP-MD 19	1.85 (1.34)	1–6	17.0	item deleted ^a^
SToP-MD 20	2.95 (1.37)	1–6	39.0	item deleted ^b^
SToP-MD 21	3.73 (1.57)	1–6	54.6	
SToP-MD 22	3.37 (1.40)	1–6	47.4	

^a^ = item deleted because of low item difficulty

^b^ = item deleted because negative item polarity

#### Factor structure

The Kaiser–Meyer–Olkin measure (KMO) of sampling adequacy indicated good suitability of the data for factor analysis (KMO = 0.86), and Bartlett’s test of sphericity was significant (χ²(45, *N* = 266) = 1116.55, *p* < 0.001), indicating adequate inter-item correlations.

An exploratory factor analysis using principal axis factoring with oblimin rotation was conducted. Factor retention was additionally evaluated using parallel analysis based on polychoric correlations. Parallel analysis suggested up to five factors. However, the additional factors beyond the two-factor solution had very small eigenvalues and did not yield interpretable or conceptually coherent item clusters. Therefore, a two-factor solution was retained on the basis of interpretability, parsimony, and theoretical consistency with the intended stigma dimensions.

The final solution comprised two factors: “Prejudiced Stigmatization” (SToP-MD-PS; eigenvalue = 3.98), which explained 39.78% of the variance, and “Assumption of Problems” (SToP-MD-AP; eigenvalue = 1.07), which explained 10.70% of the variance. Together, the two factors accounted for 50.48% of the total variance. As shown in Table 3, most factor loadings were moderate to high, supporting the interpretability of the two-factor solution [52]. One item (Item 4) showed a comparatively lower loading (0.31) but was retained due to its theoretical relevance and acceptable simple structure.

**Table 3:** Factor loadings of the finally included items of the Stigma Towards People with Mental Disorders Scale (SToP-MD) based on an exploratory factor analysis with principal axis factoring and oblimin rotation (Study 1; *N* = 266)

Item #	Factor loadings	
	PS	AP
SToP-MD 1	0.779	
SToP-MD 3		0.493
SToP-MD 4	0.312	
SToP-MD 8	0.683	
SToP-MD 9	0.453	
SToP-MD 12	0.497	
SToP-MD 14	0.592	
SToP-MD 18		0.418
SToP-MD 21		0.443
SToP-MD 22	0.521	

*PS* Prejudiced Stigmatization, *AP* Assumption of Problems

#### Scale properties

Internal consistency was assessed using McDonald’s ω. The SToP-MD-PS subscale with 7 items had an ω of 0.83, thus the internal consistency of the scale is good, whereas the SToP-MD-AP subscale with 3 items had an ω of 0.51, indicating limited internal consistency. The possible sum score of the SToP-MD-PS subscale ranges from 7 to 42. In contrast, the answers of participants in this study ranged from 8 to 42, with a mean of 20.30 (*SD* = 7.13) and the possible sum score of the SToP-MD-AP subscale ranges from 3 to 18, whereas answers of participants in this study ranged from 4 to 18, with a mean of 11.50 (*SD* = 3.00). The Kolmogorov-Smirnov-test showed that both SToP-MD subscale sum scores in this population were not normally distributed (*p* < 0.001). A more detailed view of the data of the SToP-MD-PS subscale showed a skewness of 1.08, which indicates an asymmetrical distribution with a long tail to the right.

In contrast, the kurtosis of 1.96 indicates a more peaked distribution than a Gaussian distribution. Contrarily, data of the SToP-MD-AP subscale showed a skewness of -0.20, which means an asymmetrical distribution with a long tail to the left. In contrast, the kurtosis of -0.15 indicates a less peaked distribution with lighter tails than a Gaussian distribution. The inter-item-correlations for items of the SToP-MD-PS subscale ranged from 0.48 to 0.62, and for items of the SToP-MD-AP subscale from 0.29 to 0.35. Both SToP-MD subscales showed significant association with a medium effect size (*r*_*s*_ = 0.432, *p* < 0.001). The psychometric properties of the SToP-MD subscales and the other measures used in this study are presented in Table 4.

**Table 4:** Characteristics of data of used measurements

	M	SD	Min	Max	ω	skewness	kurtosis	K-S-test
SToP-MD-PS	20.30	7.13	8	42	0.84	1.08	1.96	*p* < 0.001
SToP-MD-AP	11.50	3.00	4	18	0.51	-0.20	-0.15	*p* < 0.001
SDI	26.59	6.28	7	35	0.92	-1.07	1.13	*p* < 0.001
DSS	48.15	11.25	19	90	0.86	0.66	1.80	*p* = 0.001
DSS-PS	17.76	7.37	9	45	0.87	1.42	2.20	*p* < 0.001
DSS-PC	30.38	6.80	10	45	0.85	-0.46	0.31	*p* = 0.001
NEO-FFI-N	32.08	8.16	12	56	0.87	0.45	0.11	*p* < 0.001
NEO-FFI-A	45.12	6.74	27	59	0.82	-0.57	0.10	*p* < 0.001
NEO-FFI-O	44.30	6.92	21	59	0.78	-0.27	0.21	*p* = 0.018
ATDP	94.32	13.83	54	125	0.88	-0.32	-0.35	*p* = 0.010
ATDP-D	67.13	12.06	37	90	0.87	-0.31	-0.50	*p* = 0.006
ATDP-R	27.20	3.77	12	36	0.68	-0.54	0.79	*p* < 0.001
BIDR	79.28	12.76	48	117	0.67	0.14	-0.10	*p* = 0.040
BIDR-S	41.42	7.75	20	64	0.66	0.18	-0.16	*p* = 0.200
BIDR-O	37.87	8.88	14	63	0.63	-0.21	-0.05	*p* = 0.048
CCSS	41.10	13.19	17	87	0.85	0.66	0.35	*p* = 0.001
CCSS-S	24.72	8.33	8	48	0.80	0.16	-0.52	*p* = 0.067
CCSS-I	8.66	4.09	5	30	0.79	2.30	7.15	*p* < 0.001
CCSS-R	7.73	4.13	4	23	0.71	1.40	1.84	*p* < 0.001

*ω* McDonald’s omega, *K-S-test* Kolmogorov-Smirnov-test, *SToP-MD* Stigma towards people with mental disorders scale, *SToP-MD-PS* prejudiced stigmatization subscale, *SToP-MD-AP* assumption of problems subscale, *ATDP* Attitudes toward physically disabled persons, *ATDP-D* discomfort and insecurity in the presence of physically disabled persons subscale, *ATDP-R* rejection of social integration subscale, *SDI* Social Distance Items, *NEO-FFI* NEO five factor inventory, *NEO-FFI-N* neuroticism subscale, *NEO-FFI-A* agreeableness subscale, *NEO-FFI-O* open to experience subscale, *DSS* Depression Stigma Scale, *DSS-PS* personal subscale, *DSS-PC* perceived subscale, *BIDR* Balanced Inventory of Desirable Responding, *BIDR-S* self-deception subscale, *BIDR-O* other-deception subscale, *CCSS* Cognitions Concerning Suicide Scale, *CCSS-S* right to commit suicide subscale, *CCSS-I* interpersonal gesture subscale, *CCSS-R* resiliency subscale

#### Construct validity

Spearman’s correlation analyses were conducted to examine associations between the SToP-MD subscales and external measures (see Table 5). Both subscales showed strong positive correlations with measures of SDI and DSS, particularly DSS-PS. Negative associations were found with openness to experience and agreeableness (NEO-FFI). Most correlations with CCSS and BIDR were non-significant, whereas small correlations were found with CCSS-I and the BIDR total score.

**Table 5:** Spearman’s correlation coefficients between SToP-MD subscales and external measures (Study 1; *N* = 266)

	SToP-MD-PS		SToP-MD-AP	
	*r*_s_	*p*	*r*_s_	*p*
SDI	**0.626**	**< 0.001**	**0.456**	**< 0.001**
DSS	**0.587**	**< 0.001**	**0.430**	**< 0.001**
DSS-PS	**0.715**	**< 0.001**	**0.539**	**< 0.001**
DSS-PC	**0.218**	**< 0.001**	0.120	0.050
NEO-N	0.010	0.874	0.107	0.082
NEO-O	**− 0.225**	**< 0.001**	**− 0.135**	**0.028**
NEO-A	**− 0.242**	**< 0.001**	**− 0.328**	**< 0.001**
ATDP	**− 0.382**	**< 0.001**	**− 0.270**	**< 0.001**
ATDP-D	**− 0.370**	**< 0.001**	**− 0.257**	**< 0.001**
ATDP-R	**− 0.166**	**0.007**	**− 0.145**	**0.018**
CCSS	0.047	0.447	**0.174**	**0.004**
CCSS-S	− 0.069	0.265	0.050	0.414
CCSS-I	**0.139**	**0.024**	**0.226**	**< 0.001**
CCSS-R	0.029	0.642	0.116	0.060
BIDR	− 0.029	0.642	**− 0.141**	**0.021**
BIDR-S	− 0.015	0.809	− 0.078	0.205
BIDR-O	− 0.023	0.705	− 0.116	0.058

*SToP-MD-PS* prejudiced stigmatization subscale of the Stigma toward People with Mental Disorders scale (SToP-MD), *SToP-MD-AP* assumption of problems subscale, *SDI* Social Distance Items, *DSS* Depression Stigma Scale (total score), *DSS-PS* DSS personal stigma, *DSS-PC* DSS perceived stigma, *NEO-N/O/A* Neuroticism, Openness, and Agreeableness subscales of the NEO Five Factor Inventory, *ATDP* Attitudes Toward Disabled Persons (total score), *ATDP-D* discomfort and insecurity in presence of physically disabled persons, *ATDP-R* rejection of social integration, *CCSS* Cognitions Concerning Suicide Scale (total score), *CCSS-S/I/R * CCSS subscales (right to commit suicide/interpersonal gesture/resiliency), *BIDR* Balanced Inventory of Desirable Responding (total score), *BIDR-S/O* BIDR self- and other-deception subscales, *r*_*s*_ Spearman’s rho, *p* significance level

Correlations with *p* < 0.05 are considered statistically significant and are shown in bold

#### Demographic variables

The results of the Χ^2^-tests revealed a significant positive association between the SToP-MD-PS score and participants’ gender (*η* = 0.417, adjusted *η*^2^ = 0.039, *p* < 0.001). In contrast, there was no significant association between the SToP-MD-AP score and participants’ gender (*η* = 0.307, adjusted *η*^2^ = 0.010, *p* = 0.057). Conversely, the SToP-MD-AP score showed a positive significant association with participants’ age (*r*_*s*_ = 0.167, *p* = 0.006). In contrast, the SToP-MD-PS score was not significantly associated with participants’ age (*r*_*s*_ = 0.108, *p* = 0.079).

Additionally, there were significant associations between the SToP-MD subscales and participants’ educational background (SToP-MD-PS: *η* = 0.307, adjusted *η*^2^ = 0.375, *p* = 0.011; SToP-MD-AP: *η* = 0.307, adjusted *η*^2^ = 0.276, *p* = 0.043).

## Discussion

The study investigated the factor structure, reliability, and construct validity of the newly developed Stigma Towards People with Mental Disorders (SToP-MD) scale.

First, we tested the adequacy of all 22 items initially included in the questionnaire. The item selection led to the elimination of 12 items: 9 due to low item difficulty and 3 due to negative item polarity. Thus, the final version of the SToP-MD scale consisted of 10 items. It is striking that almost all items with a negative polarity (i.e., requiring reverse scoring) were eliminated. There is evidence that the polarity of items fundamentally impacts the dimensionality of measures [53, 54]. Three of these items with a negative polarity additionally had low item discrimination scores (items 2, 17, 20), whereas item 7 was excluded because of low item difficulty. The polarity of these items could be associated with the item difficulty or item discrimination. Future research could examine whether rephrasing these items in positive polarity would yield improved psychometric properties.

Furthermore, five additional items with positive polarity were excluded due to low item discrimination (items 11, 13, 15, 16, 19). A closer evaluation of these items leads to the conjecture that these items are not adaptable in a stigma scale that is built to assess stigmatization towards persons with mental disorders in general (e.g., item 11: “It is best just to get out of the way of people with mental disorders” or item 15: “Many people with mental disorders are less intelligent”). Furthermore, it could be possible that participants do not agree with such statements in general but would agree with them related to specific mental disorders. Thus, it is possible that participants would differentiate between, for instance, a person with major depression or a person with schizophrenia when answering items like “Mental disorders are often a sign of a weakness of character” or “It is best, just to go out of the way of people with mental disorders”. However, this assumption remains untested in the current study.

An exploratory factor analysis based on principal axis factoring indicated a two-factor structure. The number of the remaining items and the extent of factor loadings indicated a reliable interpretation of both subscales. However, the SToP-MD-AP subscale showed a limited internal consistency and should therefore be interpreted cautiously. This is also reflected in the items’ focus, which captures many aspects of stigmatization. Future research should consider expanding and refining this dimension before drawing firm conclusions about its structural stability. While the sample size of this study was adequate for the statistical methods used, it remains to be seen whether the factor structure identified in the present study will be reproducible in future studies with larger sample sizes.

Regarding the scale’s psychometric properties, the construct validity was supported by mostly expected associations between the SToP-MD subscales and a set of relevant criterion measures, such as social distance and depression-related stigma. As expected, higher social distance scores were significantly associated with higher SToP-MD scores. Social distance is a concept that has been used in several studies before to measure stigmatizing attitudes towards persons with mental disorders [7, 26, 32, 55–62]. According to Angermeyer and Matschinger [32], social distance can be adequately used to assess attitudes towards people with mental disorders. Furthermore, the DSS as a well-used valid and reliable stigma scale related to depression [14, 29, 30, 63, 64] was also significantly associated to the SToP-MD sum score. According to the idea of the SToP-MD, which aims to assess stigmatizing attitudes of respondents toward people with mental disorders, the association with the personal stigma subscale of the DSS was substantially stronger than with the perceived stigma subscale. This pattern of correlations supports the conceptual alignment of the SToP-MD scale with public attitudes, providing preliminary support of convergent validity, particularly concerning public stigma.

In terms of personality traits, two of the three NEO-FFI subscales—openness to experience and agreeableness—showed significant negative associations with both SToP-MD subscales. These results are consistent with prior findings indicating that lower openness and agreeableness are associated with higher levels of stigmatizing attitudes [24, 65–67]. For example, Canu et al. [68] found that agreeableness was positively linked to more favourable appraisals of adults with ADHD, and McCrae et al. [69] observed similar associations with stigma toward individuals with physical disabilities.

Contrary to our expectations, neuroticism was not significantly associated with either SToP-MD subscale. This aligns with previous findings suggesting that neuroticism may be unrelated to public stigma [70], while its role may be more pronounced in self-stigma or internalized negative beliefs [71, 72]. Taken together, these results further support the convergent validity of the SToP-MD scale by replicating known associations between specific personality traits and stigmatizing attitudes.

Discriminant validity was tested using measures of socially desirable responding, attitudes towards suicide, and attitudes towards physically disabled people. Contrary to our expectations, both SToP-MD subscales were negatively correlated with the ATDP total score and its subscales [38, 39]. This indicates that participants who endorsed more stigmatizing attitudes towards people with mental disorders reported less discomfort or rejection concerning physically disabled persons. While unexpected, this pattern may suggest that stigmatizing attitudes towards people with mental and physical impairments are not part of a general prejudice factor, but rather reflect distinct constructs. This supports the discriminant validity of the SToP-MD scale. It is also consistent with previous literature showing that stigmatization of mental illness tends to be more pronounced than that of physical illness [7, 58, 73–75].

As expected, there were no significant associations between the SToP-MD-PS and the CCSS total score or its subscales measuring the right to commit suicide (CCSS-S) and resiliency (CCSS-R) [41, 76]. However, a significant positive correlation was found with the CCSS subscale for suicide as an interpersonal gesture (CCSS-I), albeit with a small effect size. The same was true for the SToP-MD-AP, which additionally showed weak associations with the overall CCSS score. One explanation may be that certain items in the CCSS-I (e.g., “Taking my own life would be a good way to make sure I would always be remembered”) are interpreted by participants as indicators of psychological instability, leading to associations with stigmatizing attitudes. Prior research has shown that even professionals sometimes misclassify suicidal behaviour as a symptom of mental illness [76]. Nevertheless, since the majority of CCSS dimensions did not correlate with SToP-MD subscales, these findings generally support discriminant validity.

Finally, and in line with our expectations, no significant associations were observed between the SToP-MD-PS and the BIDR [42, 43]. Only the SToP-MD-AP showed a small negative correlation with the BIDR total score, which may reflect slightly more openness in reporting critical views. Still, the overall weak associations suggest that the SToP-MD scale is not strongly influenced by social desirability, supporting its use in self-report formats.

The analysis of demographic variables revealed several associations with stigmatizing attitudes towards people with mental disorders. Consistent with previous research [14, 15, 77–81], female participants scored significantly lower on the SToP-MD-PS subscale than male participants. In contrast, the SToP-MD-AP subscale did not show a statistically significant difference between genders, although the trend pointed in the same direction.

Regarding age, a significant positive correlation was found only for the SToP-MD-AP subscale, suggesting that older participants were more likely to assume people with mental disorders experience persistent difficulties. The SToP-MD-PS subscale, however, did not correlate significantly with age, which is partly consistent with earlier studies showing nuanced or disorder-specific associations with age [80, 82].

Both SToP-MD subscales showed significant associations with educational background; lower levels of education were associated with stronger stigmatizing attitudes [77, 81]. Higher education was associated with lower stigma scores.

Interestingly, no significant correlation was found between income and stigma, which deviates from some prior research suggesting that lower income is associated with higher stigmatizing attitudes [26, 83]. However, this result must be interpreted cautiously given that a large proportion of the sample were students, who typically report low income regardless of their socioeconomic status. Thus, income in this sample may not accurately reflect participants’ social or educational resources. Altogether, the demographic analyses support previous findings and highlight gender, age, and education as meaningful factors in shaping stigmatizing attitudes, while also underscoring the importance of sample composition in interpreting such effects.

Using the SToP-MD scale, stigma towards people with mental disorders can be validly and reliably assessed. Beyond that, results also indicated interesting associations between stigma towards people with mental disorders and personality traits as well as stigma towards physically disabled people. These results could be seen as an indication that there are divergent constructs underlying stigmatizing behavior. Thus, future studies could investigate in more detail why some people stigmatize people with mental disorders, whereas others stigmatize physically disabled persons.

## Study 2: confirmatory factor analysis

While Study 1 provided initial evidence for the factorial structure of the SToP-MD through exploratory analyses, a crucial next step in the validation process is to test the proposed factor model in an independent sample. Study 2, therefore, aimed to examine the factor structure of the SToP-MD using confirmatory factor analysis (CFA). This approach allows for a rigorous evaluation of the hypothesized measurement model and provides insights into the dimensional stability and construct validity of the scale. By validating the factor structure in a separate sample, this study aims to further establish the psychometric robustness of the SToP-MD.

### Methods

#### Participants and procedure

The second study aimed to investigate the factor structure of the SToP-MD scale identified in Study 1. Data were collected via SoSciSurvey [23]. The procedure was largely identical to that of Study 1: participants were recruited online, completed the survey anonymously, and provided informed consent before participating.

#### Statistical analyses

We conducted a Confirmatory Factor Analysis (CFA) using the lavaan package [version 0.6–20; [84]] in R [version 4.5.2; [50]]. The weighted least squares mean and variance adjusted (WLSMV) estimator was applied to account for the ordinal nature of the data. This robust estimator accounts for non-normality by adjusting both test statistics and standard errors. The model specified two latent variables: SToP-MD-PS and SToP-MD-AP. The use of WLSMV yields more reliable fit indices and parameter estimates even under mild deviations from multivariate normality assumptions.

To evaluate model fit, we extracted several commonly used goodness-of-fit indices: the relative chi-square (χ²/*df*), the root mean square error of approximation (RMSEA) along with its 90% confidence interval, the comparative fit index (CFI), the Tucker–Lewis index (TLI), and the standardized root mean square residual (SRMR). Cut-off criteria were based on established recommendations. A χ²/*df* ratio < 3 was considered indicative of good model fit, with values < 5 interpreted as acceptable [85–87]. RMSEA values < 0.05 indicated good fit, and values between 0.05 and 0.08 were regarded as reasonable [88]. For the CFI and TLI, values > 0.90 were interpreted as evidence of good fit [89, 90]. SRMR values < 0.09 were also considered indicative of good model fit [89].

### Results

#### Sample characteristics

The complete sample of study 2 consisted *N* = 448 participants, of which 65.8% (*n* = 295) were female and 34.2% (*n* = 153) were male. The age of participants ranged from 18 to 78 years, with a mean of 28.76 years (*SD* = 10.74). Of all 448 participants, 262 (58.5%) were students, 93 (20.8%) workers/employees, 22 (4.9%) freelancers, 20 (4.5%) trainees, 12 (2.7%) clerks, 11 (2.5%) retired participants, 11 (2.5%) job-seeking participants, and 7 (1.6%) pupils and 10 (2.2%) participants had another kind of employment status.

#### Confirmatory factor analysis

A confirmatory factor analysis using the WLSMV estimator was conducted to evaluate the factorial validity of the SToP-MD scale. The proposed two-factor structure showed acceptable fit according to conventional indices (CFI = 0.97, TLI = 0.96, SRMR = 0.07), with more conservative robust fit indices indicating moderate model fit (robust CFI = 0.91, robust TLI = 0.88, robust RMSEA = 0.13). All items loaded significantly on their intended factor (standardized loadings = 0.42–0.77). The two latent factors, Prejudiced Stigmatization (PS) and Assumption of Problems (AP), were strongly correlated (*r* = 0.83).

**Fig. 1:**
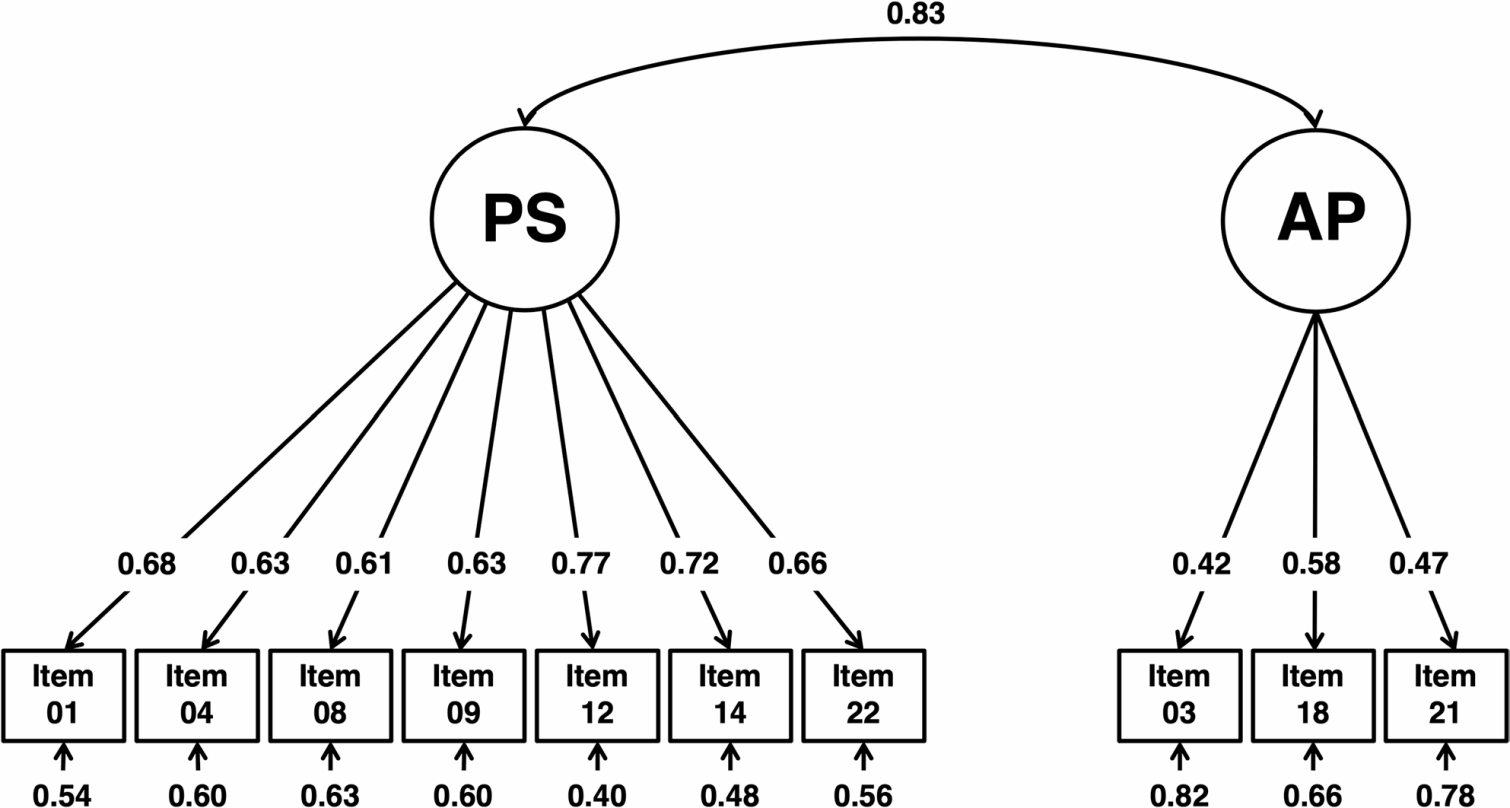
Path diagram of the two-factor confirmatory factor analysis (CFA) model for the SToP-MD scale using WLSMV estimation

Figure 1 illustrates the CFA model. The two latent variables (SToP-MD-PS and SToP-MD-AP) are represented as circles, each with directed arrows pointing to their respective observed indicators. A bidirectional arrow between the latent variables indicates their estimated correlation (*r* = 0.83), suggesting a substantial association between the two stigma dimensions.

In summary, the CFA replicated the proposed two-factor structure at the level of factor loadings identified in the exploratory analyses, although the strong association between the factors suggests that further refinement of the AP dimension may be warranted.

## Discussion

The CFA confirmed the two-factor structure identified in the EFA, with all items exhibiting meaningful, statistically significant loadings. The use of the WLSMV estimator ensured appropriate modeling of the ordinal item structure. However, the robust fit indices indicated only moderate model fit, and the latent factors were strongly correlated (*r* = 0.83). This suggests that prejudiced evaluations and problem-related assumptions are closely related aspects of public stigma, although they remained empirically distinguishable. Given the small number of items in the AP subscale, future work should consider expanding this dimension to improve measurement precision.

## Study 3: investigation of media on stigma towards people with mental disorders

Mass media are a major source of public knowledge about mental illness and play a central role in shaping stigma. Research has shown that media reports frequently associate mental disorders with violence, unpredictability, or incompetence, thereby reinforcing negative stereotypes, whereas recovery-oriented portrayals can reduce stigma [91, 92].

This dynamic was evident in the media response to the 2015 Germanwings plane crash, where the co-pilot’s presumed depression was often framed as a primary cause of the tragedy. An analysis of German print media found that more than 60% of articles implied a direct link between mental illness and the crash, frequently without adequate psychiatric context [27]. Such portrayals may reinforce misconceptions and increase public support for exclusionary attitudes. Similar effects have been observed in the context of suicide reporting, where media messages can shape attitudes toward both suicide and individuals perceived to be mentally ill [41].

Against this background, Study 3 was designed to evaluate the SToP-MD scale’s sensitivity to change. Using a brief experimental design, participants were exposed to either stigmatizing or destigmatizing media content about mental illness. By comparing SToP-MD scores before and after exposure, the study aimed to determine whether the scale can capture short-term shifts in stigmatizing attitudes, a key requirement for intervention research.

### Methods

#### Participants

A total of *N* = 269 individuals consented to participate in the study. However, three subjects (*n* = 3) discontinued participation after randomization, so data from 266 individuals (*n* = 266) were ultimately included in the analysis. No participant failed the video attention check (i.e., answered one or more of the control questions incorrectly), so all participants were included in the final analyses.

Participants were recruited via university mailing lists, social media platforms, and online psychology forums. Participation was voluntary and anonymous. All participants provided informed consent before beginning the study.

The final sample consisted predominantly of students and young adults, with a mean age of *M* = 29.45 (*SD* = 12.87; range = 18–85) years. The gender distribution was *n* = 180 (67.5%) female and *n* = 86 (32.5%) male. Participants were randomly assigned to one of the three experimental groups (positive: *n* = 92, neutral: *n* = 87, negative: *n* = 87), with approximately equal group sizes.

No significant differences were observed in age, gender, or educational background between the groups, indicating that randomization was successful. The majority of participants reported having no formal training in psychology or psychiatry.

#### Design and procedure

The study employed a between-subjects experimental design with three experimental conditions comprising positive, neutral, and negative media content related to depression. Participants were informed that they would view one of several videos, but were not informed about the study hypotheses or the specific emotional framing of the conditions. Each group was shown a short video differing in its emotional framing of depression: a positive video emphasizing recovery through psychotherapy from the German Alliance against Depression (1:24 min), a neutral educational video on neurotransmitters and depression (1:31 min), or a negatively framed news segment from Tagesschau reporting on the co-pilot’s depression in the context of the Germanwings plane crash (1:36 min).

Following the video presentation, participants first completed a brief manipulation-check questionnaire consisting of three evaluative items rated on 7-point Likert scales. These items assessed the portrayal of people with mental disorders in the video, the mood induced by the video, and the overall emotional atmosphere. Participants were then asked to provide an overall rating of the video’s emotional valence on a separate 7-point scale ranging from 1 = very negative to 7 = very positive. These items were not part of the SToP-MD. Finally, participants completed the SToP-MD questionnaire to assess stigmatizing attitudes toward people with mental disorders. The three videos were comparable in length and general presentation, whereas differences in emotional framing and content were intentional.

Finally, participants were debriefed about the purpose of the study. They were also informed that they could request the deletion of their data afterward if they disagreed with its use; however, no participant exercised this option.

#### Power analysis

An a priori power analysis was conducted using G*Power [[93], version 3.1; [94]] to determine the required sample size for detecting differences between the three experimental conditions. Based on the study design, a small to medium effect size (*f* = 0.19) was assumed, following recommendations from previous research on stigma and media exposure [e.g., [91]]. The significance level was set to α = 0.05, the statistical power to 1 – β = 0.80, and the number of groups to three.

#### Statistical analyses

To evaluate the effectiveness of the experimental manipulation, descriptive statistics (means and standard deviations) were calculated for participants’ ratings of (1) the portrayal of people with mental disorders, (2) the mood induced by the video, and (3) the emotional atmosphere of the video.

To assess group differences in stigmatizing attitudes as measured by the SToP-MD subscales, non-parametric tests were employed due to violations of normality assumptions. Specifically, a Kruskal–Wallis *H* test was used to examine overall differences across the three experimental groups (positive, neutral, negative). In the case of a significant omnibus result, pairwise Mann–Whitney U tests were conducted as post-hoc comparisons, with the Bonferroni correction applied to adjust for multiple testing (α = 0.05 / 3 = 0.016).

For all tests, effect sizes (*η*²) were calculated to quantify the magnitude of between-group differences. The threshold for statistical significance was set at *p* < 0.05 (adjusted where necessary).

All statistical analyses were conducted using R [version 4.5.2; [50]].

### Results

#### Attention check

Participants’ attentiveness to the video content was assessed through three comprehension questions per condition. Overall, no participant gave more than two incorrect answers, indicating a generally high level of attention across all groups.

In the positive condition, all participants correctly identified the video’s reference to depression (*n* = 92, 100%) and that the expert shown at the end was female (*n* = 92, 100%). Additionally, 97.8% correctly recognized the video’s focus on recovery from illness (*n* = 90).

In the neutral condition, all participants correctly identified the female physician (*n* = 87, 100%). Furthermore, 98.9% correctly answered questions regarding neurotransmitters as neural messengers (*n* = 86) and the mention of depression (*n* = 86).

In the negative condition, the majority correctly recalled that the video covered the crash in the French Alps (*n* = 86, 98.9%) and the search for the black box (*n* = 86, 98.9%). A lower proportion correctly identified the news anchor as female (*n* = 72, 82.8%).

Given these results, all participants were retained for further analysis, as none failed the attention check in a manner that would warrant exclusion based on predefined criteria.

#### Manipulation check

To assess whether the video stimuli differed in terms of perceived content and emotional impact, participants were asked to evaluate three aspects after watching their assigned video: (1) the portrayal of people with mental disorders, (2) the mood induced by the video, and (3) the overall emotional atmosphere. As expected, the videos elicited distinctive evaluations across conditions, consistent with the intended manipulation.

Regarding the portrayal of people with mental disorders, participants who viewed the positive video rated the depiction most favourably (*M* = 5.59, *SD* = 1.30), followed by the neutral (*M* = 3.98, *SD* = 0.86) and negative video (*M* = 3.00, *SD* = 1.00).

In terms of mood induction, participants exposed to the positive video reported the most positive affective response (*M* = 5.20, *SD* = 1.32), compared to the neutral (*M* = 3.93, *SD* = 1.01) and negative condition (*M* = 2.41, *SD* = 0.91).

Similarly, the emotional atmosphere was rated as most positive in the positive video condition (*M* = 5.15, *SD* = 1.24), followed by the neutral video condition (*M* = 3.68, *SD* = 0.99) and the negative video condition (*M* = 2.93, *SD* = 1.08).

These findings confirm that the videos differed significantly in emotional tone and evaluative framing, thereby validating the experimental manipulation.

#### Group differences

The results of the Kruskal–Wallis H test revealed significant rank differences between groups for the SToP-MD-PS subscale (Χ²(2) = 10.971, *p* = 0.004; *η*² = 0.034), whereas no significant differences were found for the SToP-MD-AP subscale (Χ²(2) = 2.716, *p* = 0.257; *η*² = 0.003).

Post hoc Mann–Whitney U tests, conducted to examine pairwise group differences on the SToP-MD-PS subscale, were adjusted using the Bonferroni correction (*α* < 0.016). These analyses revealed a significant difference between participants who viewed the negative video and those who viewed the positive video, with higher stigma scores in the former group (U(92, 87) = 2872.5, *p* < 0.001; *η*² = 0.059). However, no significant differences were found between the negative and neutral conditions (*U*(87, 87) = 3134.5, *p* = 0.050; *η*² = 0.022), nor between the positive and neutral conditions (U(92, 87) = 3526.0, *p* = 0.169; *η*² = 0.011).

## Discussion

This study aimed to examine the sensitivity to change of the SToP-MD scale in response to different types of media content related to depression. The findings indicate that the SToP-MD-PS subscale (Prejudiced Stigmatization) is responsive to short-term media exposure: participants who viewed a negatively framed news report displayed significantly higher stigma scores than those who watched a positively framed video. These results suggest that the SToP-MD-PS subscale is capable of detecting subtle, experimentally induced shifts in individual stigma, demonstrating its validity as a dynamic measure of attitude change.

This result aligns with prior evidence that media content plays a central role in shaping public attitudes toward mental illness. Corrigan et al. [91] showed that different narrative framings in news media can either increase or reduce stigma, depending on whether the story emphasizes recovery or reinforces fear-based stereotypes. In line with this, the current study found that exposure to negative news coverage—linking depression to a violent incident—led to significantly elevated public stigma scores, while recovery-oriented content was associated with more favourable attitudes.

These findings are further supported by von Heydendorff et al. [27], who analysed German media coverage of the Germanwings plane crash. They found that many reports overemphasized the co-pilot’s depression as a causal factor, contributing to public misperceptions of people with mental disorders as dangerous or unpredictable. The elevated SToP-MD-PS scores in the negative condition of the present study reflect how such framing can trigger measurable increases in stigma.

Moreover, the current results resonate with Biblarz et al. [41], who demonstrated that media representations of suicide and mental health can influence not only emotional responses but also cognitive evaluations of mental illness. Taken together, these studies support the conclusion that the SToP-MD scale, particularly the SToP-MD-PS subscale (Prejudiced Stigmatization), is sensitive enough to detect changes arising from brief and ecologically valid media exposure.

Interestingly, the perceived stigma subscale (SToP-MD-AP) did not show significant differences between conditions, suggesting that individual perceptions of societal attitudes may be more resistant to immediate change or less influenced by isolated media input. This highlights the differential responsiveness of the two subscales and supports the use of the SToP-MD as a multidimensional tool.

Overall, the findings underline the importance of the SToP-MD as a change-sensitive instrument, capable of capturing context-dependent fluctuations in stigmatizing attitudes. This makes it a promising tool for evaluating stigma-reduction interventions or experimental manipulations, particularly those involving media stimuli.

## General discussion

The present series of studies aimed to develop and validate the Stigma Towards People with Mental Disorders (SToP-MD) scale, a multidimensional instrument designed to assess stigmatizing attitudes toward individuals with mental health conditions. Across three studies, we examined the scale’s factorial structure, psychometric properties, and sensitivity to short-term changes following media exposure. Taken together, the findings provide preliminary support for the SToP-MD as a brief and conceptually grounded measure of public stigma.

Study 1 established the initial factor structure of the scale using exploratory factor analysis. The results suggested a two-factor solution comprising Prejudiced Stigmatization (SToP-MD-PS) and Assumption of Problems (SToP-MD-AP). The PS subscale demonstrated good internal consistency and meaningful associations with established measures of stigma and social distance, supporting convergent validity.

The AP subscale showed limited internal consistency, indicating heterogeneity among the included items. This suggests that the construct captured by this dimension may not yet be sufficiently well-defined. Future research should therefore aim to refine and expand this subscale. Given these limitations, interpretations based on the AP subscale should be made with caution. It may also be worthwhile to examine whether a more parsimonious one-factor solution focusing on the PS dimension is sufficient.

Study 2 provided a confirmatory test of the two-factor structure in an independent sample using CFA with an estimator appropriate for ordinal data. All items loaded significantly on their intended factors, and conventional fit indices indicated acceptable model fit. However, robust fit indices suggested only moderate fit, and the two latent factors were strongly correlated. This pattern indicates substantial overlap between the dimensions, suggesting that prejudiced evaluations and assumptions about long-term burden represent closely related aspects of public stigma rather than entirely independent constructs. Nevertheless, the two-factor model remained empirically distinguishable and conceptually interpretable.

Study 3 examined the sensitivity of the SToP-MD to experimentally induced changes in stigmatizing attitudes following exposure to different types of media content. The results demonstrated that the PS subscale was responsive to negative versus positive framing of depression, supporting its utility as a dynamic measure of short-term attitudinal shifts. In contrast, the AP subscale did not show significant changes across conditions, suggesting that beliefs about the long-term implications of mental illness may be more stable or less susceptible to immediate situational influences. This differential responsiveness further supports the interpretation of the two subscales as related but functionally distinct components of stigma.

Across all studies, several consistent patterns emerged. First, the PS dimension showed stronger reliability, clearer validity evidence, and greater responsiveness to contextual influences than the AP dimension. Second, demographic variables such as gender and education were associated with stigma levels in expected directions, replicating prior findings. Third, associations with personality traits and social distance measures further supported the construct validity of the PS subscale, while discriminant validity was partially supported for the AP dimension.

Overall, the SToP-MD scale offers a brief and theoretically informed instrument for assessing stigmatizing attitudes toward people with mental disorders. While the PS subscale demonstrated solid psychometric properties, the AP subscale requires further development to enhance reliability and structural stability. Future research should replicate the factor structure in more diverse and representative samples, explore potential item expansion for the AP dimension, and examine the scale’s predictive and longitudinal validity. Such work will clarify whether the two-factor structure reflects stable dimensions of stigma or whether a more parsimonious representation may ultimately prove more appropriate.

In sum, the present findings provide preliminary support for the SToP-MD as a useful tool in stigma research, public health monitoring, and intervention evaluation, while highlighting important directions for further refinement.

### Limitations

Several limitations have to be considered when interpreting the current results. First, since 69.9% of the sample was female and 100% were Caucasian, future research should investigate the use of the scale in a more diverse population. Second, only the German version of the SToP-MD scale was used in this study. Consequently, the results of this study need to be replicated in versions for other languages. Third, the sample was relatively young, with a mean age of 28.77 years, and 57.1% of the participants were students. Thus, the factorial structure has to be confirmed in older samples and samples that are more representative of the general population.

Additionally, the mean income of participants was relatively high, with a concomitantly high educational level. Thus, the psychometric properties of the scale in other samples is highly recommended. Fourth, data for this study were exclusively collected via an online survey. While equivalence of online and paper-pencil has been shown [95–97], it is possible that a paper-pencil version of the SToP-MD scale shows different psychometric properties. Fifth, the scale only assesses stigmatizing attitudes towards people with mental disorders, but neglects differentiation of public and self-stigma [2, 9, 98, 99]. Sixth, although Study 3 provided initial evidence for short-term sensitivity to change, we did not investigate the test–retest reliability of the SToP-MD scale. Future studies should therefore examine the temporal stability of the scores in the absence of intervention as well as their sensitivity to meaningful attitudinal change over longer periods. Seventh, we used the term “mental disorder” exclusively in the questionnaire. As studies showed, psychosocial and biogenetic explanations and labeling are preferred and associated with lower stigmatization or prejudice [100, 101]. Thus, future studies should investigate whether there are differences in answers when the items refer to other terms like “mental illness”, “mental distress”, or “mental crisis” instead of “mental disorders”.

## Conclusion

We conclude that the current study provides preliminary evidence for the utility of the Stigma Towards People with Mental Disorders Scale (SToP-MD). The SToP-MD scale is a brief, economical, self-report measure with good internal consistency and a reasonable number of items to assess stigmatizing attitudes towards people with mental disorders. Reliable instruments with clear psychometric properties for assessing stigmatizing attitudes toward people with mental disorders are rare [1], and many existing scales do not simultaneously capture prejudiced evaluations such as dangerousness or unpredictability and assumptions about persistent personal and societal burden. The scale described in the present study could be a helpful tool to assess stigmatizing attitudes towards people with mental disorders in the population, which, for instance, can be an indicator for the usage of anti-stigma campaigns or the provision of further educational information material. However, the construct validity of the subscales warrants further investigation in future studies with more heterogeneous samples.

Nevertheless, the SToP-MD scale is based on appropriate data analysis and demonstrates preliminary evidence of validity and acceptable internal consistency. Considering that it can be used to assess stigma towards people with mental disorders in general, consists of only 10 items – and thus it can be evaluated without additional expenditure of time – it provides a good supplement to already established stigma scales.

## Supplementary Information

Supplementary Material 1.

Supplementary Material 2.

## Data Availability

All datasets used and analyzed during the current study will be freely available at https://osf.io/fh7cw.

## Abbreviations

AQ-27: Attribution Questionnaire (27–item version)
ATDP: Attitudes Toward Disabled Persons scale
ATDP-D: Discomfort and Insecurity subscale
ATDP-R: Rejection of Social Integration subscale
BIDR: Balanced Inventory of Desirable Responding
BIDR-O: Other–Deception subscale
BIDR-S: Self–Deception subscale
CAMI: Community Attitudes toward the Mentally Ill Inventory
CCSS: Cognitions Concerning Suicide Scale
CCSS-I: Interpersonal Gesture subscale
CCSS-R: Resiliency subscale
CCSS-S: Right to Commit Suicide subscale
CFA: Confirmatory Factor Analysis
CFI: Comparative Fit Index
df: Degrees of freedom
DSS: Depression Stigma Scale
DSS-PC: Depression Stigma Scale–Perceived Stigma subscale
DSS-PS: Depression Stigma Scale–Personal Stigma subscale
EFA: Exploratory Factor Analysis
EKB: Einstellung gegenüber Körperbehinderten
KMO: Kaiser–Meyer–Olkin measure
M: Mean
NEO-FFI: NEO Five–Factor Inventory
NEO-FFI–A: Agreeableness subscale
NEO-FFI–N: Neuroticism subscale
NEO-FFI–O: Openness to Experience subscale
OMI: Opinions about Mental Illness scale
OMS-HC: Opening Minds Scale for Health Care Providers
OMS-WA: Opening Minds Workplace Attitudes Scale
PPMI: Prejudice towards People with Mental Illness scale
RMSEA: Root Mean Square Error of Approximation
r_s_: Spearman’s rank correlation coefficient
SD: Standard deviation
SDI: Social Distance Index
SRMR: Standardized Root Mean Square Residual
SSMIS: Self–Stigma of Mental Illness Scale
SToP-MD: Stigma Towards People with Mental Disorders
SToP-MD–AP: Assumption of Problems subscale
SToP-MD–PS: Prejudiced Stigmatization subscale
TLI: Tucker–Lewis Index
WLSMV: Weighted Least Squares Mean and Variance adjusted estimator
α: Cronbach’s alpha
η²: Eta squared
ω: McDonald’s omega
χ²: Chi–square statistic
