# Supplementary material for: Attitudes towards people with mental disorders: results of a psychometric evaluation and confirmatory factor analysis of the stigma towards people with mental disorders (SToP-MD) scale
Source: BMC Psychol. 2026 May 1;14:643. doi: 10.1186/s40359-026-04627-x (PMC13134182; doi:10.1186/s40359-026-04627-x)
Supplement: Supplementary file 1 — Supplementary Material 1. [file 40359_2026_4627_MOESM1_ESM.pdf]

Below you will now find several statements about people with mental disorders. Please indicate how strongly you agree with each statement. Use the scale from 1 = “completely disagree” to 6 = “completely agree”.

		1	2	3	4	5	6
1	In my opinion it would be better if people with mental disorders could only work in jobs with less responsibility.	☐	☐	☐	☐	☐	☐
2	A family member with a mental disorder often poses a great burden to the family.	☐	☐	☐	☐	☐	☐
3	People with mental disorders are burdensome for society.	☐	☐	☐	☐	☐	☐
4	One should protect others by reconsidering the non-disclosure obligation of psychotherapists and psychiatrists.	☐	☐	☐	☐	☐	☐
5	People with mental disorders often cause disproportionately high costs for the health system.	☐	☐	☐	☐	☐	☐
6	People with mental disorders are often unpredictable.	☐	☐	☐	☐	☐	☐
7	People with mental disorders have more problems following social rules.	☐	☐	☐	☐	☐	☐
8	Someone who has once suffered from a mental disorder, is likely to always have problems with it.	☐	☐	☐	☐	☐	☐
9	If I were to develop a mental disorder, I would probably conceal it from others.	☐	☐	☐	☐	☐	☐
10	People with mental disorders can cause fear in others.	☐	☐	☐	☐	☐	☐
